# Identifying the Micro-relations Underpinning Familiarity Detection in Dynamic Displays Containing Multiple Objects

**DOI:** 10.3389/fpsyg.2017.00963

**Published:** 2017-06-13

**Authors:** Jamie S. North, Ed Hope, A. Mark Williams

**Affiliations:** ^1^Expert Performance and Skill Acquisition Research Group, School of Sport, Health, and Applied Science, St. Mary’s University, TwickenhamTwickenham, United Kingdom; ^2^School of Sport and Exercise Science, Faculty of Science, Liverpool John Moores UniversityLiverpool, United Kingdom; ^3^Department of Health, Kinesiology, and Recreation, College of Health, University of Utah, Salt Lake CityUT, United States

**Keywords:** expertise, pattern recognition, perception, memory

## Abstract

We identified the important micro-relations that are perceived when attempting to recognize patterns in stimuli consisting of multiple dynamic objects. Skilled and less-skilled participants were presented with point light display sequences representing dynamic patterns in an invasion sport and were subsequently required to make familiarity based recognition judgments in three different conditions, each of which contained only a select number of features that were present at initial viewing. No differences in recognition accuracy were observed between skilled and less-skilled participants when just objects located in the periphery were presented. Yet, when presented with the relative motions of two centrally located attacking objects only, skilled participants were significantly more accurate than less-skilled participants and their recognition accuracy improved further when a target object was included against which these relative motions could be judged. Skilled participants can perceive and recognize global patterns on the basis of centrally located relational information.

## Introduction

The ability to perceive and recognize patterns between features is critical in allowing humans to function and interact in a range of activities. It is most apparent in allowing people to interact socially as they quickly and effortlessly recognize patterns between facial features to judge whether others are familiar or strangers (Want et al., 2003) and to judge affect and emotion (Bassilli, 1978, 1979). Also, pattern recognition is considered a critical component in more specialized avenues of human endeavor such as diagnostic imaging (Nodine and Kundel, 1987), computer programming (Vessey, 1987), and business (McKelvie and Wiklund, 2004) as it has been found to differentiate between high and low level performers in these domains.

The ability to perceive patterns is considered an important process and a defining characteristic of experts in domains where performers are required to make decisions and anticipate future events (Smeeton et al., 2004; Abernethy et al., 2005). The seminal research by de Groot (1946/1965) revealed that whereas Grandmaster chess players could recall positions of chess pieces with near perfect accuracy after only brief exposure to boards, less-skilled players were unable to do so. Chase and Simon (1973) subsequently replicated this finding but reported that when chess pieces were arranged randomly, the expert advantage was lost and memory performance was no different to that observed for less-skilled players. The results indicated the experts did not possess superior generic memory *per se*, but rather as a result of extended practice they developed domain specific knowledge structures which underpinned their expertise in the particular domain (Ericsson et al., 2009).

These domain specific knowledge structures, referred to as “chunks” (Chase and Simon, 1973), “templates” (Gobet and Simon, 1996), and “retrieval structures” (Ericsson and Kintsch, 1995) in different theories of expertise, are believed to be comprised of several individual items which are connected or related to each other. It is proposed that cognitive knowledge structures are developed through extended engagement within a domain and repeated exposure to performance scenarios. As a result, through their development over time, these knowledge structures allow attention to become attuned to the most important stimulus features while disregarding irrelevant information, meaning experts can reduce the complexity of a display, enabling information to be encoded more quickly, thereby facilitating pattern recognition. In contrast, novices do not possess the same sophisticated cognitive structures to guide their attentional and perceptual processes, meaning they can be easily overwhelmed by the complexity of a display and their ability to recognize patterns is impaired (Bilalic et al., 2010).

Performers such as expert chess players or elite soccer players are faced with displays that comprise of multiple objects, events, and an almost infinite number of functional relations, all of which compete for visual attention (Chun, 2000). It is argued that these performers are able to use attentional mechanisms, directed by cognitive knowledge structures, to prioritize and select only information that is relevant, meaning only a small part of the visual scene is selected and encoded to build more elaborate memory traces (Chun, 2000; Hollingworth and Henderson, 2000; Didierjean and Marmeche, 2005). Royer et al. (2015) support these assertions and challenge the popular view that faces are perceived, encoded and recognized as ‘wholes’ (see DeGutis et al., 2013; Richler and Gauthier, 2014). Using a ‘bubbles technique,’ Royer et al. (2015) were able to restrict the amount of visual information presented to participants and demonstrated that the more expert someone was at recognizing faces the fewer facial features they needed to see in order to make successful recognition judgments. Similarly, by recording eye movements, Bilalic et al. (2010) showed that expert chess players only fixate on a select few pieces, whereas novices fixate on almost every single individual feature. It appears that experts’ knowledge guides their perceptual processes and means they selectively attend to and encode only the most critical information.

The demands and challenges performers face are magnified when they must recognize patterns in dynamic and time constrained tasks (e.g., military aviation, crowd control, and sports such as soccer and ice-hockey) since the information presented differs from one moment to the next. Given the presence of absolute and common motion, and the fact that relations between features will be continually changing, it opens up the possibility that the processes which underpin pattern recognition may be different in dynamic contexts to those in static, self paced tasks (e.g., chess). Dittrich’s (1999) interactive encoding hypothesis provides an explanation for how humans recognize continually evolving stimuli involving dynamic interactions between multiple features. The motion information from, and between, display features are initially proposed to be encoded using ‘bottom–up’ low level processes before this information is then matched to an internally stored semantic template using higher order ‘top–down’ processes. A similar proposal has been made by Wong and Rogers (2007) in their recognition of temporal patterns theory in which they argue for a pre-processing stage, during which only the necessary information for pattern classification is extracted, before this information is subsequently matched to a known template in memory. Support for this initial phase comes from research which demonstrates that humans are incredibly adept at perceiving biological motion when presented with point light displays (PLDs) (see Mather and Murdoch, 1994; Shim et al., 2004; Jastorff et al., 2006). Moreover, the observation of an expert advantage in perceiving biological motion (see Ward et al., 2002) implies a role for higher order ‘top–down’ processes, which aligns with earlier explanations that suggested extended practice results in highly specialized domain-specific knowledge structures.

Williams et al. (2006) provided evidence that relational information between display features is used to inform pattern recognition in soccer. Skilled and less-skilled participants were presented with film displays showing dynamic patterns of play and later required to make recognition judgments to film and PLD stimuli. The PLD condition removed all surface level information (e.g., uniform color, postural and form cues from players, environmental conditions) and only retained information about the positions and movements of players. Skilled players demonstrated a recognition advantage in both film and PLD conditions and their recognition performance was relatively unaffected in the PLD condition. With reference to Wong and Rogers’s (2007) theory, it appears that during the initial pre-processing stage, skilled individuals encode relational information between display features, as proposed by Dittrich (1999), while the skill advantage supports the role for a higher level cognitive process which matches this information to a stored semantic template.

It appears crucial for skilled participants to pick up relational information when perceiving patterns (Gauthier and Tarr, 2002; Maurer et al., 2002; Williams et al., 2006; North et al., 2009, 2011). North et al. (2009) recorded eye movement data while participants viewed and recognized patterns of play in soccer and reported that the information conveyed by central attacking players seemed particularly important to skilled recognition. As reported by Bilalic et al. (2010) in chess, it appears that skilled performers in dynamic contexts (such as soccer) can direct their attention to a select few display features. It is less clear whether this information emerged as a function of information conveyed through the positions of players at an isolated point in time or through motion information (relative motion between players or absolute motion of an individual player). Williams et al. (2012) presented skilled and less-skilled participants with dynamic film displays showing multiple independent, yet interacting, features before making recognition judgments to three different types of stimuli, namely, dynamic, static, and random. Dynamic stimuli contained relational and motion information, whereas static stimuli showed only the final frame of a sequence for the same duration of time as dynamic clips which maintained relational but removed motion information. Finally, the random stimuli presented each individual frame from a dynamic sequence in a random order so that the same amount of information was presented but relational and motion information were disrupted. Skilled participants demonstrated a recognition advantage for dynamic stimuli only, suggesting the low level information that is extracted before matching to a semantic template is encoded on the basis of motion information.

The dynamic stimuli used by Williams et al. (2012) contained both absolute and relative motion, meaning it was not possible to determine whether the initial ‘bottom–up’ perceptual processes were based on extracting either one or both of these sources of information. Dittrich and Lea (1994) originally argued that either motion information of isolated independent features (i.e., absolute motion) *or* interactions between various independent features (i.e., relative motion) could be encoded. In a second experiment, Williams et al. (2012) had skilled and less-skilled participants complete recognition tasks to normal dynamic stimuli and mirror-reversed dynamic stimuli which maintained relative motions between features but altered the absolution motion of each individual feature. The skill advantage was observed across both normal dynamic stimuli and mirror reversed stimuli, suggesting it is solely relative motion between features that is initially encoded in the pattern recognition process and not absolute motion.

Wong and Rogers (2007) suggest that the fundamental challenge is for researchers to identify the minimal set of features which enable accurate pattern recognition. In the current study, we extend recent research by examining the information that is perceived when recognizing dynamic patterns. In addition to the ‘global pattern’ (i.e., the interactions between all display features) that exists in a display, more localized ‘micro patterns’ (i.e., interactions between smaller numbers of localized display features) are also present. Some researchers (see Bilalic et al., 2010; Royer et al., 2015) have suggested that skilled individuals encode these ‘local micro-relations’ to perceive and recognize the ‘global pattern.’ We sought to examine this issue further by looking at whether participants were capable of recognizing global patterns (comprising of multiple features) on the basis of localized patterns between limited numbers of display features, and whether certain localized patterns were more important than others when recognizing structure and familiarity.

We first presented participants with a series of dynamic PLD stimuli showing multiple individual objects that represented players interacting in a structured invasion game. In the recognition phase, stimuli were edited to produce three different recognition conditions, each showing different micro patterns. In Condition 1, only the positions and movements of two peripheral display features was presented with all other information being occluded. In Condition 2, we presented the positions and movements of only the two central attacking players, whereas in Condition 3 the positions and movements of the two central attacking players along with the ball and player in possession was presented. Given published reports involving visual search (North et al., 2009) and spatial occlusion methods (Williams et al., 2012, see Experiment 3) which have indicated central features to be of particular importance, we hypothesized that skilled participants would demonstrate superior recognition performance in Conditions 2 and 3 compared to Condition 1. Furthermore, given the apparent importance of an organizational or target cue against which other features can be encoded against (cf. Dittrich and Lea, 1994; North et al., 2009), we hypothesized that skilled participants would demonstrate superior recognition performance in Condition 3 compared with Condition 2.

## Materials and Methods

### Participants

A total of 10 skilled (*M*age = 21.8 years, *SD* = 2.2) and 13 less-skilled (*M*age = 20.9 years, *SD* = 2.6) soccer players participated. Participants were considered skilled if they had previously played at an English Premier League Academy and/or were currently playing at a semi-professional standard. Skilled participants had been playing soccer competitively for an average of 12.9 years (*SD* = 3.1). In contrast, participants classed as less-skilled had not participated in soccer above a recreational/amateur level. Less-skilled participants reported an average of 10.7 years (*SD* = 3.1) participation. All participants reported normal or corrected to normal levels of visual function, provided written informed consent, and were free to withdraw from the experiment at any stage. Ethical approval was granted by the institution where data collection took place (ethics approval number: 09/SPS/010).

### Test Films

Participants were presented with two separate test films; an initial viewing phase test film followed by a recognition phase test film. All the stimuli used in viewing and recognition test films were presented in PLD format. The stimuli all represented action sequences in the sport of soccer that was originally filmed from a raised position (approximate height 9 m) behind the goal (approximate distance 15 m) using a tripod-mounted camera (Canon XM-2, Tokyo, Japan). The camera did not pan or zoom and its position ensured that information from wide areas of the display was not excluded. All clips used in the experiment were considered ‘structured’ as rated by three independent expert soccer coaches. Two of the coaches were licensed by the Football Association and had over 5 years coaching experience at a semi-professional level. The other coach had extensive coaching experience (over 10 years) at professional and international level and held the highest level coaching award offered by the Football Association. Each clip was rated using a Likert-type scale from 0 to 10 (0 being very low in structure and 10 being very high in structure) with structured clips considered as those which were most representative of typical attacking patterns and sequences. Only clips with a mean rating of seven or above were used in the experiment. This replicates the methods used to judge structure used by Williams et al. (2006, 2012) and North et al. (2009, 2011).

**FIGURE 1:**
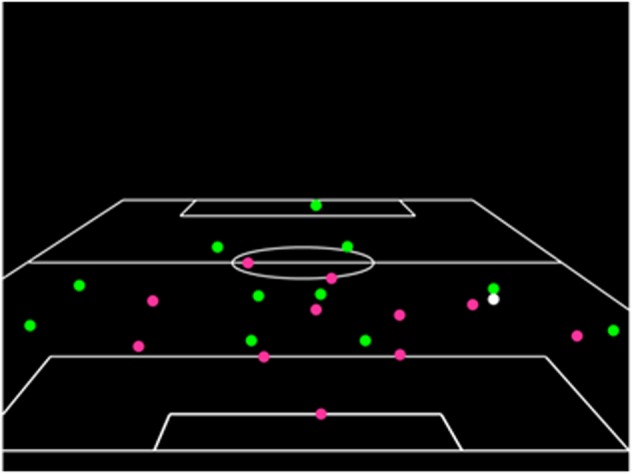
A schematic illustration of the information presented during the viewing phase.

The viewing phase test film contained 18 individual action sequences, each of which was 5-s in length and showed players represented as points of light moving against a black background inside an outline of the pitch. The attacking team was represented as green points of light, the defending team as red points of light, and the ball was white. Each clip showed a developing attacking sequence that finished when the player in possession of the ball was about to make an attacking pass. For an illustration of the information presented in the viewing phase, see **Figure 1**. There was a 5-s inter-trial interval between the conclusion of one clip and onset of the next.

**FIGURE 2:**
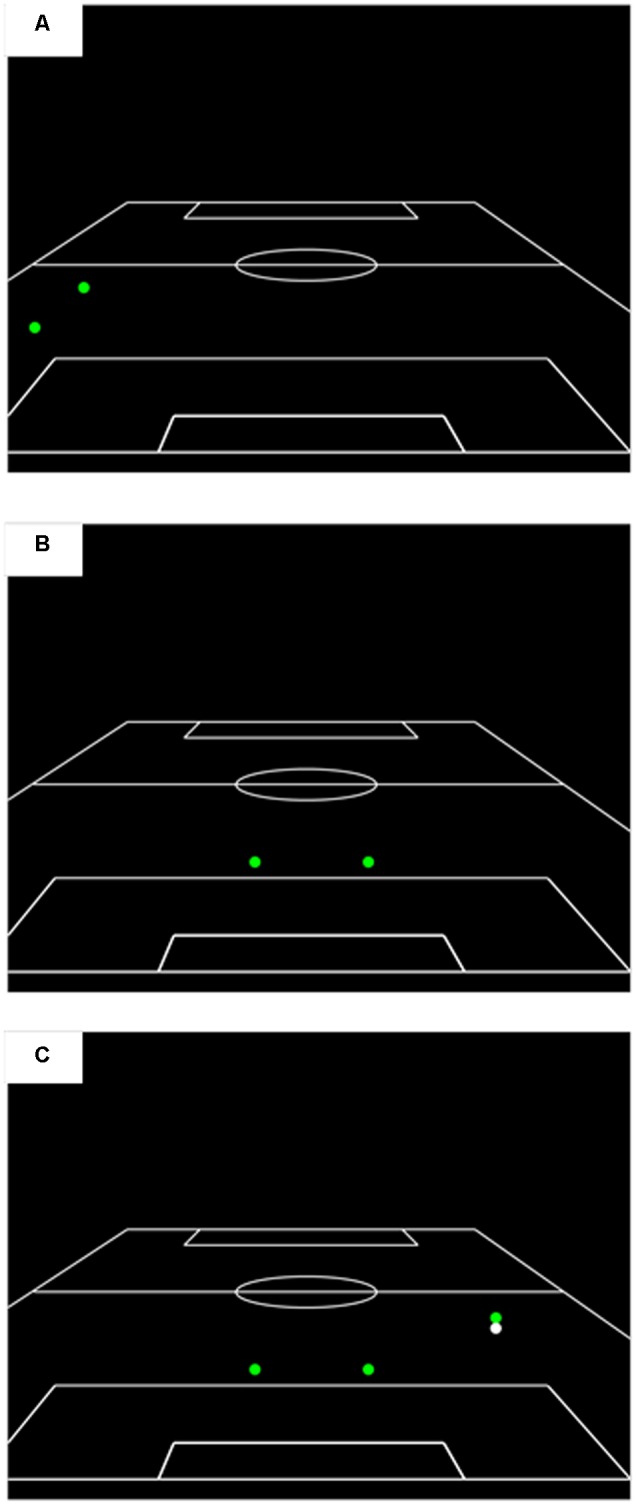
A schematic illustration of the information presented during the recognition phase in **(A)** peripheral players only, **(B)** central offensive players only, and **(C)** central offensive players plus ball and player in possession conditions.

The recognition phase test film also contained 18 action sequences, 12 of which had been presented previously in the viewing phase and 6 that were novel. All the clips presented in the recognition phase had been edited so as to isolate the relative motion information between specific features by occluding all other display features. Specifically, the recognition phase test film contained three separate conditions. Condition 1 contained only two peripheral features that were far away from the focus of the action contained in the clip. Typically, these two peripheral features represented defensive players for the attacking team that were in the opposite half of the pitch to the ball/action area. Condition 2 presented only the two central offensive features from the attacking team. Condition 3 presented the two central offensive features from the attacking team as well as the player in possession of the ball and the ball. Examples of the information presented in each of these conditions are shown in **Figure 2**. The recognition phase contained six clips from each of these conditions, which is comparable to the number of clips per condition in previous research investigating pattern recall and pattern recognition (six trials per condition, Abernethy et al., 2005; North et al., 2009; eight trials per condition, Smeeton et al., 2004). For each subset of six clips, four were edited versions of clips that had been presented in the viewing phase and two were edited versions of clips that had not been presented previously in the experiment. Video clips in the recognition phase were presented in a randomized order that was kept consistent across participants. As in the viewing phase, clips in the recognition phase were 5-s in length with a 5-s inter-trial interval between the conclusion of one clip and the onset of the next.

### Apparatus

To convert video film footage into PLD format, the original film clips were saved into “.avi” format using video editing software (Adobe Premiere, Adobe Systems Incorporated, San Jose, CA, United States). IrfanView ^1^ was used to export these clips to the software package AnalysaSoccer (Liverpool John Moores University, United Kingdom) which then allowed the positions and movements of players to be digitized and reconstructed so they were represented as points of light against a black background. Test films were presented using a DVD player (Panasonic, DMR-E50, Osaka, Japan) and projector (Sharp, XG-NV2E, Manchester, United Kingdom) with images projected onto a 9′ × 12′ screen (Cinefold, Spiceland, IN, United States) at a rate of 25 frames per second.

### Procedure

Participants were tested individually and sat in a chair 3 m from the projection screen such that the image subtended a viewing angle of approximately 40°. Prior to being shown the viewing phase film, participants were told they would be presented with a series of clips, all 5-s in length, showing PLD sequences of play in soccer that would build up to a point where the player in possession (or point of light) was about to make an attacking pass, however, each clip would occlude at the final moment before this event occurred. Participants were instructed to watch the clips as if they were a central defensive player, but were told that no specific response was required while viewing these clips.

After the viewing phase, participants were given a short break (approximately 15 min, which is comparable to that employed in previous pattern recognition research, e.g., Goldin, 1979; Williams et al., 2012) during which they completed a practice history questionnaire relating to their soccer experience and skills. Participants were then informed they would be presented with another series of action sequences, all of which were again in PLD format, but that all of the clips in the recognition phase test film had been edited so as to only show certain points of light and that all others had been removed, and that each clip was unique (i.e., no clip was replicated across different conditions). The participants were told that some of these clips were edited versions of clips that had been presented in the earlier viewing phase, whereas others were edited versions of clips that had not been presented previously. Participants were instructed to watch each clip for its duration and their task then was to make a familiarity judgment as to whether the clip was an edited version of one that had been presented in the earlier viewing phase (i.e., respond “yes”) or not (respond “no”). Participants made their responses using a pen and paper response sheet. Prior to completing the recognition phase, a familiarization procedure was employed in which participants were presented with three examples from each of the edited conditions.

### Data Analysis

Recognition accuracy was calculated by dividing the total number of correct familiarity judgments by the total number of clips presented and then multiplying by 100 to give a percentage accuracy score. Recognition accuracy was then analyzed using a mixed design two-way analysis of variance (ANOVA) in which the between participant factor was skill (skilled vs. less-skilled) and the within participant factor was display (peripheral players vs. central offensive players vs. central offensive players + player in possession). The data were tested for normality using the Shapiro–Wilks test and this assumption was satisfied. Partial eta squared values $\eta_{p}^{2}$ are provided as a measure of effect size for all main effects and interactions and Cohen’s *d* values are also reported where there are comparisons between two means. For the repeated measures variable, violations of sphericity were corrected by adjusting the degree of freedom using the Greenhouse–Geisser correction when the epsilon was estimated to be less than 0.75 and the Huynh–Feldt correction when greater than 0.75 (Girden, 1992). Any *post hoc* tests of within-group differences were conducted using Bonferroni-corrected comparisons. The alpha level for statistical significance was set as *p* < 0.05.

## Results

**FIGURE 3:**
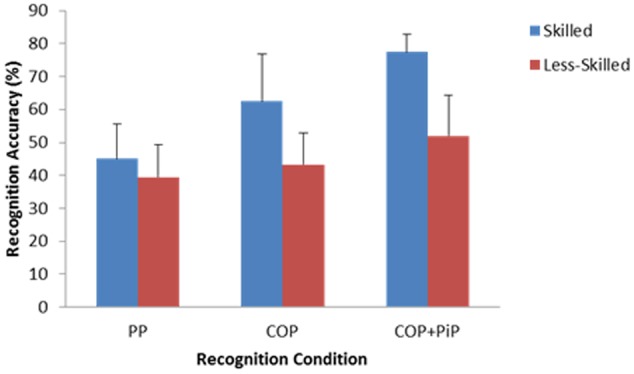
The Skill × Display interaction on anticipation accuracy (+1 SD). PP is Condition 1 (Peripheral Players only); COP is Condition 2 (Central Offensive Players only); COP+PiP is Condition 3 (Central Offensive Players + ball and Player in Possession only).

Analysis of variance revealed a significant Skill × Display interaction, *F*(1.776,37.294) = 5.67, *p* < 0.01, $\eta_{p}^{2}$ = 0.21. Skilled participants were more accurate than less-skilled participants in their recognition judgments when presented with only the movements of central offensive players (*M* = 62.50%, *SD* = 14.43 vs. *M* = 43.27%, *SD* = 9.70) and when presented with the movements of central offensive players plus the ball and player in possession of the ball (*M* = 77.50%, *SD* = 5.27 vs. *M* = 51.92%, *SD* = 12.34), *d*’s = 1.56 and 2.70 respectively. However, there was relatively little difference in recognition accuracy between skilled (*M* = 45.00%, *SD* = 10.54) and less-skilled (*M* = 39.42%, *SD* = 10.01) participants when presented with only two peripheral display features, *d* = 0.54. This interaction is illustrated in **Figure 3**.

The main effect for skill was significant, *F*(1,21) = 34.49, *p* < 0.01, $\eta_{p}^{2}$ = 0.622. Skilled participants (*M* = 61.66%, *SD* = 7.56) made more accurate recognition judgments than less-skilled participants (*M* = 44.86%, *SD* = 6.17), *d* = 2.43. Finally, there was a main effect of display on recognition accuracy, *F*(1.776,37.294) = 27.51, *p* < 0.01, $\eta_{p}^{2}$ = 0.57. Bonferroni-corrected pairwise comparisons demonstrated that participants were significantly more accurate in their recognition judgments in Condition 3 when presented with two central offensive players plus the ball and player in possession (*M* = 63.04%, *SD* = 16.20) than in Condition 2 when only presented with the two central offensive players (*M* = 51.63%, *SD* = 15.22) or in Condition 1 when only presented with two peripheral players (*M* = 41.85%, *SD* = 10.4), *p*’s < 0.05, *d*’s = 0.73 and 1.56 respectively. Recognition performance was also significantly more accurate in Condition 2 when presented with just two central offensive players than in Condition 1 when presented with two peripheral players, *d* = 0.75.

## Discussion

We examined if skilled performers could perceive and recognize global patterns in displays that contained multiple objects on the basis of localized patterns and micro relations between limited numbers of display features. Previously, researchers have presented evidence that skilled performers encode relationships between features (see North et al., 2009, 2011) as a function of relative motion information (see Williams et al., 2012) to recognize patterns. Moreover, published reports from domains such as chess (Bilalic et al., 2010), face recognition (Royer et al., 2015), and object tracking (Fehd and Seiffert, 2008) suggest that experts only need to attend to a select few display features in order to perform successfully. In light of the above findings, we were interested in testing whether the relative motion information between localized display features in dynamic stimuli was sufficient to recognize a larger pattern and, if so, whether relationships between certain display features provided more important information than other sources. We presented skilled and less-skilled soccer players with a series of PLD clips representing dynamic patterns and later asked them to complete a recognition task to stimuli that had been edited to show only the movements of, and relations between, a limited number of display features.

A Skill × Display interaction was observed, which, as hypothesized, was due to skilled participants demonstrating superior recognition accuracy than their less-skilled counterparts when presented with two central attacking objects (*d* = 1.56) and these two features as well as the ball and player in possession (*d* = 2.70), yet their recognition accuracy dropped to the level of less-skilled participants when presented with only a limited number of peripheral objects (*d* = 0.54). It has been established previously that experts process relational information between display features (see Gauthier and Tarr, 2002; Maurer et al., 2002; Williams et al., 2006, 2012). However, our findings indicate that not all sources of information and relative motion in a display are equal. It appears certain local patterns within a display’s global pattern may be redundant in the encoding process (i.e., the two peripheral objects in the displays in this experiment), whereas other local patterns are more important. Specifically, the evidence presented here suggests that in dynamic invasion sports it is the information presented in central regions of the display and potentially the micro-relations between central attacking features that are initially attended to and encoded by skilled performers, and which subsequently convey important information to successfully recognize patterns. The pre-processing stage in Wong and Rogers’s (2007) recognition of temporal patterns theory would therefore seem to focus on extracting information from within a specific area of the display and potentially the functional relations between a limited number of display features in this region (namely those positioned centrally in the display), which is subsequently sufficient to ensure accurate matching against templates stored in memory in a later phase of the pattern recognition process.

The results are in line with research findings in chess (see Bilalic et al., 2010) and face recognition (see Royer et al., 2015) which report that experts only encode a select few objects in a display and that central regions of complex displays is where gaze is typically directed (Fehd and Seiffert, 2008). It seems experts are able to utilize elaborate domain specific knowledge structures to direct attention exclusively to the most important information sources. In our specific context, it appears skilled participants’ attention was directed to the central region of the display as when they were subsequently presented with just the relations and movements between central attacking objects it was sufficient to activate the representation for the global pattern and enable successful pattern matching. However, recognition accuracy improved further still when additional information was presented from this central region (i.e., Condition 3 when the ball and player in possession was also presented), whereas when presented with movements and relations between players in peripheral positions they were unable to recognize patterns. One interpretation is that skilled players encode micro-relations between central attacking players and can subsequently utilize just this information to recognize global patterns. An alternative interpretation is that it is not necessarily these localized micro-relations that are important, but rather it is more generally information from central areas that is initially encoded and important for subsequent recognition. The finding that recognition accuracy improves as more information is presented from central areas supports this latter argument and future research should seek to disentangle if it is specific micro-relations between central features that are important or alternatively if it is amount of information from central regions that is of greater importance.

The suggestion that information conveyed by relative motions between central attacking objects provides important information to recognize global patterns is in line with previous research where visual search data have been recorded (North et al., 2009) and spatial occlusion techniques employed (Williams et al., 2006). North et al. (2009) reported that skilled participants focused a greater percentage of viewing time on the movements of these features, while Williams et al. (2006) found that recognition accuracy of skilled participants suffered when these features were selectively occluded from the display. However, limitations associated with these methods (e.g., the potential disassociation between gaze data and attentional allocation and the possibility that when using film occlusion methods form based cues as well as relational information are removed) means that by presenting solely the relative motion between these features, we have provided more direct evidence that it is the encoding of relative motion information between central attacking objects that provides important information for skilled participants to recognize patterns. Nevertheless, recognition accuracy improved further still when additional centrally located features were presented, and so an alternative interpretation is that is more generally centrally located information sources that are important (and as the amount of centrally presented information increases so too does recognition accuracy) rather than specific localized micro-relations. In future, researchers should include stimuli in which the ball is also positioned toward the periphery of the display and examine if micro-relations between central features still remain critical to pattern recognition (i.e., independent of ball location) or if micro-relations between features on the sides of the display now become more important (i.e., the critical micro-relations are dependent on ball location). This is especially pertinent given recent research findings which have demonstrated the dynamic nature of perceptual processing as a function of ball location (see Roca et al., 2013; North et al., 2016).

The main effect of display showed that not only did recognition accuracy improve from Condition 1 (two peripheral objects) to Condition 2 (two central attacking objects), but it improved further still in Condition 3 (two central attacking objects plus the ball and player in possession). By reporting a skill difference when only the two central attacking players were presented, we have demonstrated that the localized relative motions between these features provides important information to recognize patterns. However, it appears that the addition of extra features in central areas of the display (in this case the ball and player in possession) provided additional information which improved pattern recognition performance further still. Such additional information may act as a reference point against which the relational information conveyed by the central attackers can be judged. In earlier research by Dittrich and Lea (1994), participants were required to detect intentionality and biologically meaningful motion in simulated dynamic displays comprising of abstract objects (they used a set of moving letters) and they found intentionality was most easily recognized when both ‘target’ and ‘goal’ features were present. When the ‘goal’ feature was removed, although participants could still recognize intentionality, their ability to do so was impaired. The information contained in the dynamic motions of the ‘target’ feature was sufficient to perceive and recognize intentionality, yet the presence of an additional ‘goal’ feature to judge this information against enhanced observers’ ability to recognize the display. We argue that in our task the presence of localized relative motions between central attacking objects provides important information to recognize patterns, however, the addition of an extra feature provided a point of reference against which these relative motions could be organized and enhanced participants’ ability to complete the perceptual-cognitive process of pattern recognition (see also North et al., 2009). The alternative interpretation is that this extra feature simply provided more centrally located information and future research should seek to address the subtle nuances underpinning pattern recognition by distinguishing between the contributions of amount of information presented and information conveyed by specific relationships.

In summary, we have provided evidence that skilled participants initially encode information from central regions of a complex display. When presented with just localized relative motions between central attacking players this information was sufficient to recognize patterns in displays that comprise multiple independent objects. However, further work is necessary to conclusively state that it is these localized micro relations that are critical to pattern recognition processes over and above the amount of information that is presented centrally. The significant skill advantage suggests this low level bottom–up perceptual process is later completed through a top–down process in which the information that has been encoded is matched to a stored semantic template. This argument supports the proposals contained within Dittrich’s (1999) interactive encoding hypothesis as well as Wong and Rogers’s (2007) recognition of temporal patterns theory. Our findings suggest that centrally located information (and potentially localized patterns) contain important information that is necessary to recognize more global patterns. In future, researchers should look to investigate whether a domain’s environmental and task characteristics may constrain the critical perceptual information that underpins successful performance on the task.

## Ethics Statement

This study was carried out in accordance with the recommendations of Liverpool John Moores University ethics committee with written informed consent from all subjects. All subjects gave written informed consent in accordance with the Declaration of Helsinki. The protocol was approved by the Liverpool John Moores University ethics committee (ethics approval number: 09/SPS/010).

## Author Contributions

JSN, EH, and AMW were involved in study design. EH collected data. EH and JSN conducted data analyses. JSN, EH, and AMW interpreted output of data analyses. JSN, EH, and AMW all contributed to write-up of research project.

## Conflict of Interest Statement

The authors declare that the research was conducted in the absence of any commercial or financial relationships that could be construed as a potential conflict of interest.

## Footnotes

1 www.irfanview.com
